# Innovative Detection and Segmentation of Mobility Activities in Patients Living with Parkinson’s Disease Using a Single Ankle-Positioned Smartwatch

**DOI:** 10.3390/s24175486

**Published:** 2024-08-24

**Authors:** Etienne Goubault, Christian Duval, Camille Martin, Karina Lebel

**Affiliations:** 1 Institut de Recherche Robert-Sauvé en Santé et en Sécurité du Travail (IRSST), 505 Boul. de Maisonneuve O, Montréal, QC H3A 3C2, Canada; etienne.goubault@irsst.qc.ca; 2 Département des Sciences de l’Activité Physique, Université du Québec à Montréal, Montréal, QC H2X 1Y4, Canada; duval.christian@uqam.ca; 3 Centre de Recherche de l’Institut Universitaire de Gériatrie de Montréal, Montréal, QC H3W 1W6, Canada; 4 Centre de Recherche sur le Vieillissement, CIUSSS de l’Estrie—CHUS, Sherbrooke, QC J1H 4C4, Canada; camille.martin3@usherbrooke.ca; 5 Département de Génie Électrique et de Génie Informatique, Université de Sherbrooke, Sherbrooke, QC J1K 2R1, Canada

**Keywords:** activities of daily living, inertial sensors, automatic detection, movement disorders

## Abstract

Background: The automatic detection of activities of daily living (ADL) is necessary to improve long-term home-based monitoring of Parkinson’s disease (PD) symptoms. While most body-worn sensor algorithms for ADL detection were developed using laboratory research systems covering full-body kinematics, it is now crucial to achieve ADL detection using a single body-worn sensor that remains commercially available and affordable for ecological use. Aim: to detect and segment Walking, Turning, Sitting-down, and Standing-up activities of patients with PD using a Smartwatch positioned at the ankle. Method: Twenty-two patients living with PD performed a Timed Up and Go (TUG) task three times before engaging in cleaning ADL in a simulated free-living environment during a 3 min trial. Accelerations and angular velocities of the right or left ankle were recorded in three dimensions using a Smartwatch. The TUG task was used to develop detection algorithms for Walking, Turning, Sitting-down, and Standing-up, while the 3 min trial in the free-living environment was used to test and validate these algorithms. Sensitivity, specificity, and F-scores were calculated based on a manual segmentation of ADL. Results: Sensitivity, specificity, and F-scores were 96.5%, 94.7%, and 96.0% for Walking; 90.0%, 93.6%, and 91.7% for Turning; 57.5%, 70.5%, and 52.3% for Sitting-down; and 57.5%, 72.9%, and 54.1% for Standing-up. The median of time difference between the manual and automatic segmentation was 1.31 s for Walking, 0.71 s for Turning, 2.75 s for Sitting-down, and 2.35 s for Standing-up. Conclusion: The results of this study demonstrate that segmenting ADL to characterize the mobility of people with PD based on a single Smartwatch can be comparable to manual segmentation while requiring significantly less time. While Walking and Turning were well detected, Sitting-down and Standing-up will require further investigation to develop better algorithms. Nonetheless, these achievements increase the odds of success in implementing wearable technologies for PD monitoring in ecological environments.

## 1. Introduction

Parkinson’s disease (PD) is a neurodegenerative disorder characterized by both motor and non-motor symptoms, including tremor, bradykinesia, postural instability, and muscle rigidity [1]. These symptoms progressively impair motor functions in people with PD and affect their ability to perform activities of daily living (ADL) to varying degrees. Effective levodopa therapy, determined by clinicians after clinical assessment, can help manage and improve quality of life by reducing the impact of these motor dysfunctions on ADL.

Following the evolution of patients’ symptomatology in a hospital environment can be challenging for both clinicians and patients depending on the disease severity and the societal context, such as a pandemic context. In recent years, algorithms using body-worn sensors have been developed to provide real-time feedback for rehabilitation [2,3] and pharmaceutical intervention [4,5]. To improve long-term home-based monitoring of PD symptoms, it is crucial to determine patients’ ADL such as Walking, Turning, or Sitting. Indeed, characterizing the mobility of people living with PD would constitute important information about the effectiveness of medication [6,7,8]. In addition, detecting such patterns will enable the better monitoring of PD symptoms such as the freezing of gait while turning or walking straight [9] or dyskinesia while sitting [10]. While most body-worn sensor algorithms for ADL detection were initially developed using laboratory research systems covering full-body kinematics, some identified a reduced set of sensors capable of detecting ADL in patients with PD with high accuracy for better ecological use [11,12,13]. While these algorithms demonstrated high performance scores in detecting and segmenting task events with an effort to reduce the number of sensors, they still used 4 to 6 sensors positioned on different body segments to detect common gross ADL [12,14]. This number of sensors remains too high for ecological use and still raises acceptability and acceptance issues for patients with PD.

A recent review revealed that the acceptance of wearable technology for monitoring and assessing disease evolution such as PD still remains a challenge in many aspects, including ease-of-use, comfortability, size, and weight [15], although the use of three-sensor set positioned on the wrist, ankle, and hip is still preferred by patients with PD as compared to the self-reporting method [16]. Nevertheless, patients with PD do not feel fully at ease wearing the sensors in public. Patients indicated high levels of willingness to wear the three-sensor set for less than two months, but their willingness to use the system over longer time periods was lower. In accordance with these results, a recent study showed that the wearable sensors were the least well tolerated among multimodal sensors [17]. Therefore, to increase the odds of success in implementing wearable technologies for PD monitoring, it is now crucial to achieve ADL detection using a single body-worn sensor that remains commercially available and affordable for ecological use.

To answer this issue, Sun et al. [18] developed an ADL detector application based on Smartwatch inertial data. Their algorithms detected activities such as *Walking*, *Running*, *Upstairs*, and *Downstairs* based on arm swing pattern recognition. For *Sitting* detection, the authors used empirical thresholds for the mean and variance of *x*, *y*, and *z* acceleration data. These algorithms achieved over 92% accuracy in detecting *Walking*, *Running*, and *Sitting*. However, participants’ demographic characteristics were not extensively discussed in this study, which raises questions about their age and health status and thus the generalizability of the algorithms. Indeed, such algorithms could encounter challenges when applied to patients with PD, as arm swinging is significantly reduced in this population compared to healthy individuals [19]. Additionally, patients with PD often experience tremors and/or dyskinesia of varying severity in distal segments [20,21,22,23], which complicates the use of wrist Smartwatches for gross motor activity detection in characterizing the mobility of patients. This is also true for detecting sedentary activities such as Sitting, since the wrists of people with PD may move substantially with tremors and dyskinesia during sedentary behavior such as Sitting. This makes the use of such a threshold for the PD population using wrist Smartwatches impossible. More recently, the WATCH-PD study [24] used the open-source GaitPy library [25,26] to extract gait events and parameters. However, not much information was given on their validity except for *Turning*, which reached 90% sensitivity and 75% specificity [26]. Similarly, Jung et al. [27] used a machine learning algorithm fed with inertial data recorded at the lower back of 21 healthy subjects and 9 patients suffering from a neurological pathology due to a cerebral lesion or having undergone or being about to undergo an orthopaedical surgery. They classified walking phases, non-sedentary phases, and sedentary phases with 90%, 75%, and 83% sensitivity, respectively, with an overall F1-score of 75%. However, the use of the low-back sensor was reported to be less comfortable than wrist or ankle sensors and could result in less acceptability and acceptance by patients for daily life symptom monitoring [16]. Therefore, it is essential to validate event detection and segmentation algorithms using wrist or ankle sensors to improve the reliability of monitoring and increase the odds of acceptability and acceptance of sensor use in patients’ real-life environments [28].

Previous research by Mannini et al. [29] showed that ankle data provided better classification accuracy for monitoring ADL in healthy adults (95%) compared to wrist data (84.7%). Ankle-positioned inertial sensors can accurately measure foot impacts during walking [30] and may offer an alternative for ADL detection in individuals with reduced arm swinging, such as patients with PD. Therefore, the objective of this study was to develop detection and segmentation algorithms for gross motor patterns using Smartwatch inertial sensors positioned at the ankle of patients living with PD in a simulated free-living environment. We focused on the ability of the sensor to automatically ***detect*** four tasks, *Walking*, *Turning*, *Sitting-down*, and *Standing-up.* Furthermore, we assessed the ability of algorithms to ***segment*** the tasks by detecting the initiation and end of each task.

## 2. Materials and Methods

### 2.1. Participants

Twenty-two patients diagnosed with idiopathic PD according to the UK Parkinson’s Disease Society Brain Bank’s clinical diagnosis criteria [31] were recruited in this cross-sectional study through the Quebec Parkinson Network and via clinicians specialized in movement disorders. Patients having orthopaedic conditions or experiencing dementia that could hinder the execution of the required tasks were excluded from this study. One participant used her own cane during the experiment. Data collection took place at the Research Centre on Aging at the Université de Sherbrooke. The study protocol was approved by the Comité d’éthique de la recherche du CIUSSS de l’Estrie—CHUS (MP-31-2022-4265), and each participant provided informed consent. Characteristics of the patients are presented in Table 1.

### 2.2. Instrumentation

Participants were equipped with a commercial Smartwatch (Apple Watch Series 5, Apple Inc., Cupertino, CA, USA) positioned on their right or left ankle, i.e., contralateral to their most symptomatic side. The Smartwatch is equipped with a 3-axis accelerometer and gyroscope, recording signals at 50 Hz.

Additionally, participants were equipped with 39 reflective markers tracked by 17 cameras (OptiTrack, Natural Point Inc., Corvallis, OR, USA), from which the avatar was used as a gold standard for validating the detection and segmentation of ADL with the Smartwatch. These markers were carefully positioned to allow for the use of OptiTrack’s conventional Full Body model, and data were collected at 100 Hz. To minimize inter-participant variability, the same experimenter positioned the Smartwatch and the reflective markers for all participants.

Participants were also wearing a Smartwatch (Apple Watch Series 5, Apple Inc., Cupertino, CA, USA) positioned on their most symptomatic wrist and a full-body inertial measurement unit motion capture system (MTw, XSENS, Enschede, The Netherlands), but these were not considered for the subject of this paper.

### 2.3. Experimental Procedures

Upon participant arrival, the clinical part of the Unified Parkinson’s disease rating scale (UPDRS-Part III) was performed by a trained examiner for each participant who was not clinically assessed in the past two months prior to the experiment. Then, participants were equipped with the above-mentioned sensors and asked to perform the following clinical tests:

Timed Up and Go (TUG). Participants were seated on a chair and instructed to stand up, walk up to a marker on the floor (3 m away), turn around, walk back to the chair, and sit down. This task was repeated three times, and participants were instructed to walk at their own pace and to not help themselves with their arms for Standing-up and Sitting-down.

Following these clinical tests, participants were asked to perform ADL in a simulated free-living environment during 3 min, 4 min, and 5 min trials. Color-coded objects were strategically placed at various locations and heights throughout the designated space. Participants navigated the environment, collected the objects, and placed them in corresponding color-coded baskets, which also place in the space. The environment included straight walking paths (8 m), an obstacle to step over (23 cm high), left and right turns while walking, and turning around on the spot. Objects and baskets were placed at two different height levels: low-level (50–70 cm high), and mid-level (80–100 cm high). Additionally, three armless chairs were provided to simulate Sitting-down and Standing-up activities. Participants started by sitting in a designated chair, walked at a normal pace, carried one object at a time, sat in each chair at least once, stepped over the obstacle at least once during each trial, and ensured each basket had an equivalent number of items by the end of each task. A 3 min rest period was observed between each trial.

### 2.4. Detection Algorithms

Data processing and statistical analyses were conducted using Matlab R2023a (The MathWorks Inc., Natick, MA, USA). Second-order zero-lag Butterworth filters were applied to all data. The algorithms for Walking, Turning, Sitting-down, and Standing-up were developed based on the TUG task and validated in the simulated free-living environment. A rule-based approach was used in this study for developing the algorithms, which was based on the expertise of the team behind the detection and segmentation algorithms for the elderly and people with PD, using inertial measurement units [12,13,32], and they presented the advantages of being simple, fast to run, and easily reproducible.

*Walking*. For Walking detection, a threshold was first established using acceleration data obtained in a static condition, i.e., with the Smartwatch stationary on a table. After applying a 0.5 Hz high-pass filter to remove the gravity components of the signals, the activity threshold was calculated as

(1) $$Thresh_{Activity}=mean\left(vertical\;acc.\right)+30\times sd\left(vertical\;acc.\right)$$

Ankle acceleration was filtered using a 0.5–0.8 Hz band-pass filter (ABPlow) to remove gravity components and highlight the general pattern of foot impact on the ground. Walking was then identified when the pre-processed vertical acceleration (i.e., along the shank) exceeded the threshold identified with Equation (1) for at least 0.4 s. Small impacts generated during stationary steps (i.e., on place steps) were then removed from walking segments. For this, the ankle acceleration was filtered with a 0.5–3 Hz band-pass filter (ABP_walk_) to remove gravity components while keeping voluntary movement signal in a larger extent to identify smaller peaks generated during stationary steps. The range of acceleration was then calculated for each segment as

(2) $$Range_{Acc}=max\left(A_{BPwalk}\right)-min\left(A_{BPwalk}\right)$$

**Figure 1:**
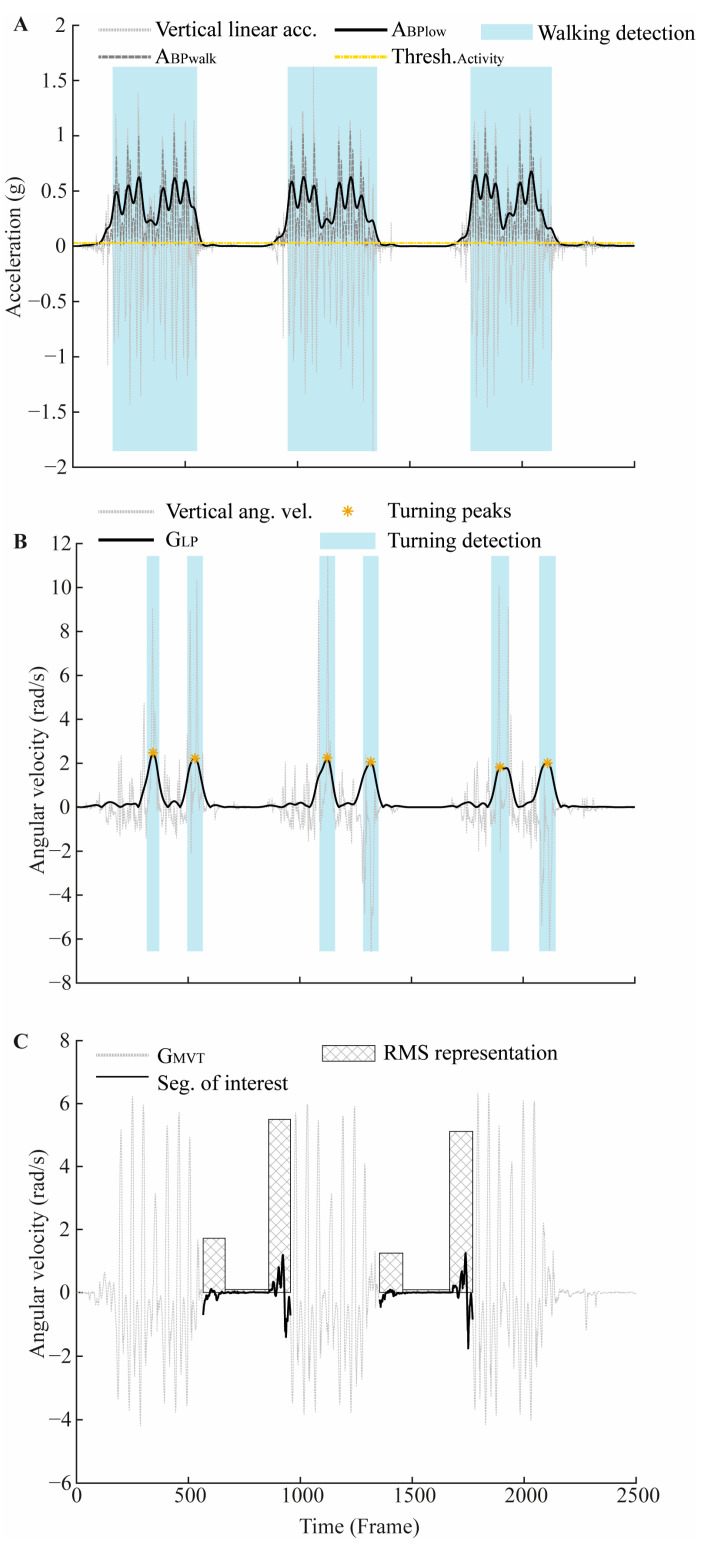
Activity detection. (**A**) Example of Walking detection using vertical linear acceleration (light grey) filtered (A_BPlow_) using a 0.5–0.8 Hz band-pass filter (black). The threshold detection is represented in yellow, while A_BPwalk_ used for refining the start and end of each walking segment is represented in dark grey. The blue areas represent the final walking segments. (**B**) Example of Turning detection using the vertical angular velocity (light grey) filtered using a 0.5 Hz low-pass filter and then rectified (G_LP_ represented in black). Yellow stars represent the peaks identified when turning. The blue areas represent the final turning segments. (**C**) Example of Sitting-down and Standing-up detections using medio-lateral angular velocity filtered using a 4 Hz low-pass filter (G_MVT_ represented in light grey). Black portions represent the segments of interest, where Sitting-down and Standing-up occur. The RMS representation shows the difference between Sitting-down, Standing-up, and the middle part corresponding to the sitting phase. The three examples were provided from the three consecutive TUG tasks of one patient.

Adjustments to walking segments were then performed by removing each foot impact–acceleration peak lower than Range_Acc._/4 from the walking segments. Additionally, walking segments spaced by less than 1 s were concatenated, and segments composed of a single foot impact–acceleration peak were also removed. Finally, the start and end of each walking segment were refined using ABP_walk_ peaks corresponding to the first and last ABP_low_ peaks of segments initially used to define walking segments. This refinement was performed because ABP_walk_ peaks are sharper than the ABP_low_ peaks (see Figure 1A).

*Turning*. Angular velocity around the vertical axis of the ankle sensor was filtered using a 0.5 Hz low-pass filter and then rectified (G_LP_). This cutoff frequency was empirically determined to highlight Turning movement pattern, as shown in Figure 1B. Peaks with a minimum peak prominence of 0.1 were initially identified. Subsequently, peaks greater than either the mean amplitude peaks identified or those with a minimum peak prominence of 0.8 were defined as turning events. The beginning and end of the Turning event were set at the peak location plus and minus half of the peak width, respectively. Finally, overlapping turning segments were concatenated (Figure 1B).

*Sitting-down/Standing-up (i.e., Stand-to-sit/Sit-to-stand transitions).* Angular velocity data of the ankle sensor around the z-axis (i.e., medio-lateral axis) were filtered using a 4 Hz low-pass filter (G_MVT_). For each non-walking segment, the root mean square (RMS) of angular velocity was calculated on the first and last 25% of the segment (RMS_F_ and RMS_L_, respectively), as well as between 25% and 75% of the segment (RMS_M_). Based on the hypothesis that angular velocity at the ankle should increase during Sitting-down and Standing-up phases compared to the sitting phase, an empirical detection threshold (Equation (3)) was set to verify if RMS_F_ and RMS_L_ were greater than RMS_M_ (Figure 1C).

(3) $$Thresh_{Sit/Stand}=RMS_{F/L}+0.33\times RMS_{F/L}$$

The complete sensor signals used for detection and segmentation are presented in Table 2.

### 2.5. Manual Segmentation

To validate the algorithms and assess detection performance, an independent examiner segmented activity during the 3 min trial using a visual full-body avatar generated from OptiTrack motion capture software. The examiner received instructions on visually marking the beginning and end of various activities during the trial, without specific markers to avoid biasing the algorithms. For Walking, the examiner was instructed to begin marking when one foot started leaving the ground and to stop when both feet were on the ground without further locomotion. Only segments of 1.5 m and more were marked. Turning was marked when one leg began a rotational movement to change direction and ended when the rotational movement ceased. Only distinct rotational movements inside a 1 m diameter were segmented; longer curves made during walking were not considered in this study. Sitting-down and Standing-up were identified by the downward and upward movements of the body from the standing and sitting position, respectively, without stipulating specific body movements. Sitting-down and Standing-up were only considered when participants remained in a sitting position for at least 2 s. Participants were instructed to perform the task at their own volition to mimic their natural movement. General guidelines were adopted to account for the variability in how participants transition from one activity to the next. For instance, some participants transitioned discretely from Standing-up to Walking, while others moved directly from Sitting-down to Walking. Therefore, the examiner had to exercise judgment in identifying these differences among participants. It took approximately 4 h to visually segment Walking, Turning, Sitting-down, and Standing-up in one 3 min trial.

### 2.6. Statistical Analyses

Sensitivity, specificity, and F-scores were calculated to evaluate the detection performance of Walking, Turning, Sitting-down, and Standing-up activities, first in the TUG task used for development and subsequently during the 3 min trial for validation. Sensitivity measures the true positive (TP) proportion, while specificity measures the true negative (TN) proportion. The F-score was defined as

(4) $$Fscore=\frac{2TP}{2TP+FN+FP}$$

where FN represents the number of false negatives, and FP denotes the numbers of false positives.

Segmentation accuracy was assessed by comparing the absolute time difference between the manual segmentation marked by the examiner for each task segment (ending–beginning) and automatic segmentation using the developed algorithm (ΔT= |Tmanual-Talgo|). A boxplot of ΔT was generated to illustrate the predictive reliability of using the algorithms to segment these activities.

## 3. Results

### 3.1. Activity Detection

Twenty-two TUG trials were used to develop the algorithms, resulting in 333 events of Walking, Turning, Sitting-down, and Standing-up activities. Walking (*n* = 112) was detected with 100% sensitivity and 71.6% specificity. Turning (*n* = 133) was identified with 100% sensitivity and 100% specificity. Sitting-down (*n* = 44) was identified with 93.2% sensitivity and 97.8% specificity. Standing-up (*n* = 44) was identified with 93.2% sensitivity and 91.7% specificity.

The confusion matrix for activity detection in the TUG trial is presented in Table 3. There was a certain degree of confusion during Walking, with the algorithms incorrectly detecting Walking while patients where actually Turning around (39.6%). Nonetheless, Turning was still correctly detected during these events, which limits the error of interpretation when looking for both results at the same time. By contrast, the confusion of Sitting-down and Standing-up during Walking were only 2.3% and 9.1%.

Twenty-two 3 min trials were used to validate the algorithms, resulting in a total of 1104 events of *Walking*, *Turning*, *Sitting-down*, and *Standing-up* activities. *Walking* (*n* = 390) was detected with 96.5% sensitivity and 94.7% specificity. *Turning* (*n* = 634) was identified with 90.2% sensitivity and 93.6% specificity. *Sitting-down* (*n* = 40) was identified with 57.5% sensitivity and 70.5% specificity. *Standing-up* (*n* = 40) was identified with 57.5% sensitivity and 72.9% specificity.

The confusion matrix for the activities is presented in Table 4. There was a significant degree of confusion during *Sitting-down* and *Standing-up* transitions, with the algorithms incorrectly detecting *Sitting-down* while patients where actually *Walking* (39.2%) or *Turning* (25.5%), and falsely identifying *Standing-up* while patients were *Walking* (28.8%) or *Turning* (37.0%). By contrast, the confusion of *Sitting-down* and *Standing-up* during Walking were only 1.0% and 0.8%, respectively, and only 0.3% and 1.1% during *Turning*.

### 3.2. Activity Segmentation

**Figure 2:**
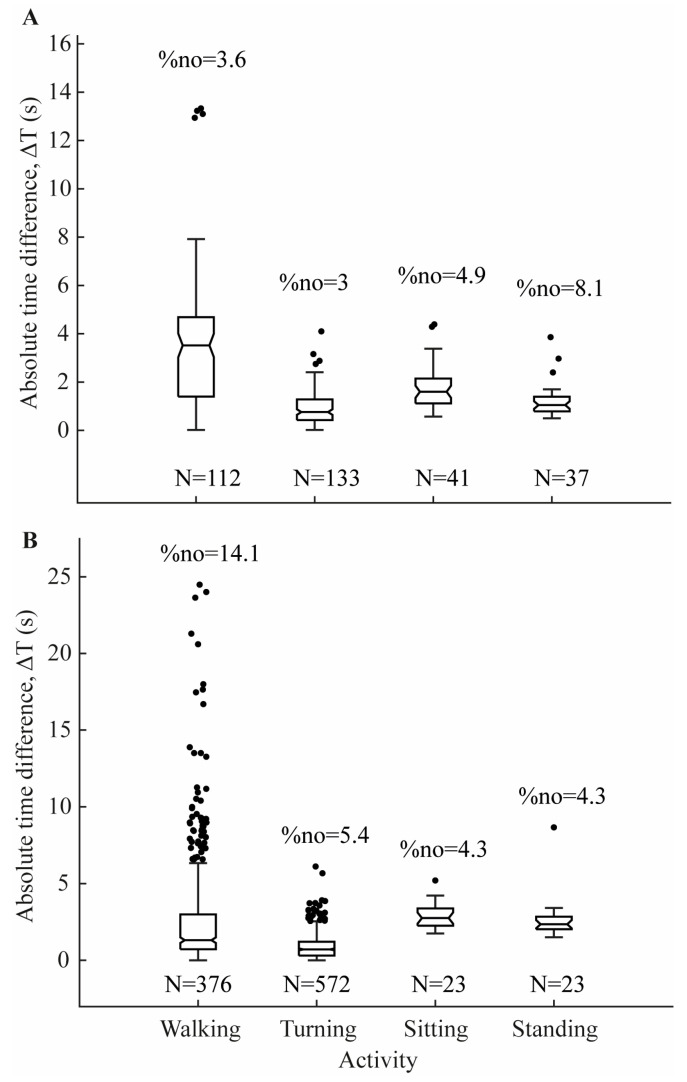
Absolute time difference (ΔT) between manual and automatic segmentations for the TUG task (**A**) and the 3 min trial (**B**). N denotes the number of task events, and %no indicates the percentage of outliers within each activity.

For the TUG task, the median time difference (ΔT) between the manual and automatic segmentation for Walking detection (*n* = 112) was 3.52 s (Figure 2A), with four outliers accounting for 3.6% of Walking events (%no). For Turning detection (*n* = 133), the median ΔT was 0.76 s, with four outliers (%no = 3.0% of events). Sitting-down detection (*n* = 41) had a median ΔT of 1.60 s, with two outliers (4.9% of events), while Standing-up detection (*n* = 37) had a median ΔT of 1.05 s, with three outliers (8.1% of events).

**Figure 3:**
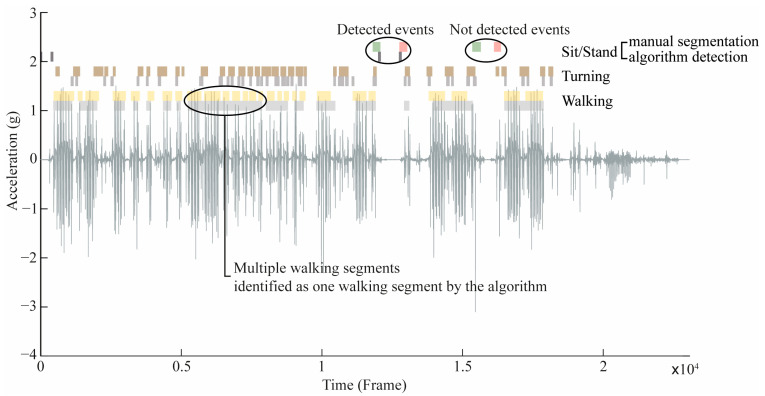
Example of 3 min segmentation. The signal represents the linear vertical acceleration. Yellow segments represent the manual Walking segmentation, while the light grey segments underneath represent the algorithm’s Walking segmentation. The brown segments represent the manual Turning segmentation, while the grey segments underneath represent the algorithm’s Turning segmentation. The green and red segments represent the manual Sitting-down and Standing-up segmentations, while the dark grey segments underneath represent the algorithm’s Sitting-down and Standing-up segmentations. The two bubbles areas on the top highlight TP (left) and FN (right) for *Sitting-down* and *Standing-up* detections. The larger bubble area highlights multiple *Walking* segments identified as one *Walking* segment by the algorithm.

For the 3 min trial, the median time difference (ΔT) between the manual and automatic segmentation for ***Walking*** detection (*n* = 376) was 1.31 s (Figure 2B), with 53 outliers accounting for 14.1% of ***Walking*** events (%no). For ***Turning*** detection (*n* = 572), the median ΔT was 0.71 s, with 31 outliers (%no = 5.4% of events). ***Sitting-down*** detection (*n* = 23) had a median ΔT of 2.75 s, with 1 outlier (4.3% of events), while ***Standing-up*** detection (*n* = 23) had a median ΔT of 2.35 s, also with 1 outlier (4.3% of events). An example of 3 min trial segmentation is shown in Figure 3.

## 4. Discussion

To the best of our knowledge, this study is the first to focus on detecting and segmenting ADL in patients with PD using only a Smartwatch positioned at the ankle. The high rates of sensitivity and specificity in detecting *Walking* and *Turning* highlight the feasibility of using a single sensor to track patient mobility in real environments. However, the moderate rates of sensitivity and specificity for *Sitting-down* and *Standing-up* indicate the need to explore other methods for detecting these specific tasks in people with PD.

Only a few studies have applied classification methods to a population of patients with PD. Jalloul et al. [14] used a set of six sensors to measure ADL such as *Walking*, *Sitting-down*, and *Standing-up* in patients with PD, achieving sensitivities of 92.4%, 88.6%, and 91.8%, respectively. However, the study’s sample size of two patients limits its generalizability. Later, Nguyen et al. [13] achieved comparable or higher sensitivities for the same ADL in a larger cohort of patients with PD (*n* = 9) and with fewer sensors (hip, thigh, trunk, and head sensors for all the ADL mentioned above). In this study, only a Smartwatch positioned at the ankles of the patients (*n* = 22) was used to detect and segment ADL. During the development phase (TUG task), Walking was detected with 100% sensitivity and 71.6% specificity (F-score = 74.2%), while during the validation phase (3 min trial), Walking was detected with 96.4% sensitivity and 94.4% specificity (F-score = 95.8%). This difference is mainly due to the nature of both tasks. During the TUG task, participants walked for 3 m before turning around to come back. Therefore, the ADL performed (Walking–Turning–Walking) are linked and accomplished without transition. The Walking segments are then very close and concatenated in one unique segment. Instead, during the 3 min trial simulating cleaning in a free-living environment, participants performed ADL more naturally without specific instructions. Walking segments are then further from each other, resulting in a better Walking detection specificity. Nonetheless, since only one Smartwatch was worn on either the right or left ankle, a small walking segment could represent varying numbers of steps depending on which leg initiated the movement. This led to potential discrepancies between manual and algorithmic detection, as a small walking segment could have been labelled as *Walking* by the examiner but removed by the algorithm due to limited detected impacts. During the TUG task, the median time difference between automatic and manual Walking segmentation was 3.52 s, with four outliers and a maximum value close to 13 s. During the 3 min trial, the median time difference between automatic and manual Walking segmentation was 1.31 s, with 53 outliers and a maximum value close to 25 s. Indeed, the automatic algorithm was set to concatenate very close walking segments [30]. Therefore, some long walking segments were manually labelled as multiple shorter segments, as shown in Figure 3, explaining the observed outliers. During the development phase, Turning was detected with 100% sensitivity and 100% specificity (F-score = 100%), with a median difference of 0.76 s to manual segmentation, while during the validation phase Turning was detected with 90.2% sensitivity and 93.6% specificity (F-score = 91.7%), with a median difference of 0.71 s to manual segmentation. During the TUG task, Turning occurred only abruptly during sharp angles of 180°. In contrast during the 3 min trial, Turning could occur abruptly during sharp angles (turn 180° or 90°) or more gradually during walking, which explains why some events were not detected using only one Smartwatch. For Sitting-down and Standing-up, the development phase showed high sensitivity (93.2%) and specificity (97.8% and 91.7%), while they were both detected with 57.5% sensitivity and 70.5% and 72.9% specificity, respectively, during the validation phase. The 3 min trial used for the validation phase was composed of multiple events not present in the TUG task used for the development phase. For instance, standing by to grab an object between two Walking segments was part of the 3 min trial, but it was not during the TUG task. Even if the approach used did not yield satisfactory results, specifically for Sitting-down and Standing-up detection, it still gave information on “sedentary” standing and sitting phases. Nonetheless, these specific results suggest the need for alternative algorithms, such as machine learning, to improve accuracy in Sitting detection for patients with PD. This will be crucial in the future for symptom monitoring in PD, particularly for assessing postural instability, freezing of gait while standing (walking straight or turning), and dyskinesia while sitting. Despite challenges in Sitting-down and Standing-up detection, the high accuracy scores in Walking and Turning detection demonstrate the potential of using commercial Smartwatches for efficient movement analysis in clinical settings for patients with PD. In addition, the simple algorithms developed can be easily reproduced for any IMU sensor positioned at the ankle for larger usability rates in clinical settings.

The results of this study demonstrate the capability of automatically classifying different activities across varying levels of symptom severity in patients with PD using only one sensor. Despite the inter-participant symptom variability, the algorithms achieved high accuracy in detecting these activities during a cleaning task performed in a simulated free-living environment. The proper segmentation of tasks was of the outmost importance in order to develop efficient algorithms to assess the quantity and quality of the task performed within those segments. Moreover, the segmentation precision was comparable to manual segmentation. In addition to ADL classification, this study is unique, as it presents the first experiment on activity segmentation in patients with PD using a single Smartwatch positioned at the ankle. This marks an important initial step in evaluating the quality of patients’ movements in their natural living environments using such technology. The next phase involves developing and validating algorithms for monitoring patients’ symptomatology in their actual living conditions. Smartwatches have the potential to detect and quantify symptom severity in patients with PD, including tremor, dyskinesia, bradykinesia, freezing of gait, and more. These tools would enable better follow-up of patients’ symptoms, quantity and quality of mobility, and treatment efficacy. Ultimately, the goal is to deploy these Smartwatches among patients with movement disorders and extract relevant clinical parameters within each activity segment to assess rehabilitation progress and pharmaceutical intervention effectiveness.

One limitation of this study is that the patients were not in the most advanced stage of the disease, with a mean Hoehn and Yahr score of 1.4 and a maximum of 2. There will be a need to challenge our algorithms with more advanced cases of PD. Nonetheless, this study marks a significant milestone in home patient monitoring. The accurate detection and segmentation of ADL in a free-living environment are essential prerequisites before developing evaluation tools for patient monitoring in their natural living settings. The outcomes of this study will prompt further analysis of patients with PD during the performance of more natural activities in a free-living environment. This will allow for a better understanding and evaluation of the effects of rehabilitation and pharmaceutical interventions. Accurate real-time assessment of performance will greatly enhance clinicians’ ability to administer corrective interventions and improve the quality of life for people living with movement disorders.

## 5. Conclusions

The results of this study demonstrated that automatic segmentation of gross activities of daily living that characterize the mobility of patients living with PD can be performed using a single Smartwatch positioned at the ankle, with comparable precision to manual segmentation, while also requiring significantly less time. This achievement is a further milestone in evaluating the quality of patients’ movement in their natural living environment, while the use of only one sensor increases the odds of acceptability by patients when implementing wearable technologies for PD monitoring in ecological environments.

## Figures and Tables

**Table 1:** Characteristics of patients.

Characteristics	Mean ± SD	Range
***Patients (n = 22, 11 Females)***		
Age (year)	66.3 ± 9.0	47–78
Weight (kg)	72.9 ± 13.6	50–97
Height (cm)	166.6 ± 8.9	152–180
Years since diagnosis	7.1 ± 5.2	1–21
Comorbidity index (/18)	4.8 ± 2.4	1–9
Moca (/30)	27.1 ± 2.8	19–30
Notthingham ADL scale (/22)	19.6 ± 1.6	16–22
***MDS-UPDRS Part III On***		
Speech (3.1)	0.4 ± 0.6	0–2
Facial expression (3.2)	0.6 ± 1.0	0–4
Neck rigidity (3.3)	0.7 ± 0.8	0–2
Arm rigidity (3.3)	0.9 ± 0.8	0–2
Leg rigidity (3.3)	0.7 ± 0.7	0–2
Finger tapping (3.4)	0.6 ± 0.7	0–2
Hand movements (3.5)	0.8 ± 0.6	0–2
Pro-sup movements of hands (3.6)	0.7 ± 0.7	0–2
Toe tapping (3.7)	0.3 ± 0.4	0–1
Leg agility (3.8)	0.3 ± 0.6	0–2
Arising from chair (3.9)	0.1 ± 0.3	0–1
Gait (3.10)	0.4 ± 0.8	0–2
Freezing of gait (3.11)	0.1 ± 0.2	0–1
Postural stability (3.12)	0.7 ± 0.8	0–2
Posture (3.13)	0.4 ± 0.5	0–1
Body bradykinesia (3.14)	0.3 ± 0.5	0–1
Postural tremor (3.15)	0.4 ± 0.6	0–2
Kinetic tremor (3.16)	0.7 ± 0.6	0–2
Rest tremor amplitude upper limbs (3.17)	0.6 ± 0.8	0–3
Constancy of rest tremor (3.18)	1.4 ± 1.3	0–4
Hoehn and Yahr score On	1.4 ± 0.5	1–2

**Table 2:** Sensor signals used to detect activities.

Task Detection	Algorithm Step	Sensor Used	Pre-Processing	Abbreviation
Walking	Threshold	Static a_z_	0.5 Hz HP filter	Thresh._Activity_
	Initial walking	Ankle a_z_	0.5–0.8 Hz BP filter	A_BPlow_
	Steps without walking	Ankle a_z_	0.5–3 Hz BP filter	A_BPwalk_
				
Turning	Initial turning	Ankle g_z_	0.5 Hz LP filter	G_LP_
				
Sitting-down/ Standing-up	RMS calculation from 1–25%, 25–75%, 75–100% of signals	Ankle g_x_, g_y_, g_z_	4 Hz LP filter	G_MVT_

**Table 3:** Activity detection confusion matrix for the TUG trial.

	Walking	Turning	Sitting-Down	Standing-Up
Walking (*n* = 390)	100	39.6	0.0	0.0
Turning (*n* = 641)	0.0	100	0.0	0.0
Sitting-down (*n* = 51)	2.3	0.0	93.2	0.0
Standing-up (*n* = 73)	9.1	0.0	0.0	93.2
F-score	74.2	100	92.1	74.5
Sen. (%)	100	100	93.2	93.2
Spec. (%)	71.6	100	97.8	91.7

Confusion matrix of the four activities detected in the TUG task as a percentage (%). *n* denotes the number of samples of each activity. The F-score represents the accuracy of activity detection. Sen. and Spec. refer to sensitivity and specificity of the algorithms, respectively. Darker shading indicates higher detection percentage.

**Table 4:** Activity detection confusion matrix.

	Walking	Turning	Sitting-Down	Standing-Up
Walking (*n* = 390)	96.5	6.4	1.0	0.8
Turning (*n* = 641)	4.9	90.2	0.3	1.1
Sitting-down (*n* = 51)	39.2	25.5	57.5	0.0
Standing-up (*n* = 73)	28.8	37.0	0.0	57.5
F-score	96.0	91.7	52.3	54.1
Sen. (%)	96.5	90.0	57.5	57.5
Spec. (%)	94.7	93.6	70.5	72.9

Confusion matrix of the four activities detected in the simulated free-living environment as a percentage (%). *n* denotes the number of samples of each activity. The F-score represents the accuracy of activity detection. Sen. and Spec. refer to sensitivity and specificity of the algorithms, respectively. Darker shading indicates higher detection percentage.

## Data Availability

The aggregated data presented in this study are available on request from the corresponding author due to ethical constraints.
